# Multiple drug resistance of *Campylobacter jejuni and Shigella* isolated from diarrhoeic children at Kapsabet County referral hospital, Kenya

**DOI:** 10.1186/s12879-021-05788-3

**Published:** 2021-01-23

**Authors:** Ongwae H. Zachariah, Mwamburi A. Lizzy, Kakai Rose, Mutuku M. Angela

**Affiliations:** 1 Department of Health Services, University of Eldoret, P.O. Box 1125, Eldoret, 30100 Kenya; 2 Department of Biological Sciences, University of Eldoret, P.O. Box 1125, Eldoret, 30100 Kenya; 3 School of Public Health and Community Development, Maseno University, Private Bag, Maseno, Kenya; 4 Department of Biological Sciences, Moi University, P.O. Box 3900, Eldoret, 30100 Kenya

**Keywords:** *Campylobacter jejuni*, *Shigella*, Diarrhoea, Antibiotic resistance

## Abstract

**Background:**

Diarrhoea is a common cause of mortality and morbidity in children under five years old. In Kenya, it has a 21% case fatality with *Enteropathogenic E. coli, Campylobacter jejuni, Shigella* spp*.* and *Salmonella spp.* accounting for 50–60% of the cases. Sulphonamides, tetracycline, ampicillin and trimethoprim/sulfamethoxazole are typically used in the treatment of diarrhoeal diseases but have become ineffective in the face of emerging antimicrobial resistance. The objective of this study was to evaluate the prevalence and antimicrobial susceptibility of *Campylobacter jejuni and Shigella* species in children under five years of age presenting with diarrhoea at Kapsabet County Referral Hospital in Kenya.

**Methods:**

Faecal samples were collected from 139 children admitted with diarrhoea. Each sample was examined macroscopically for colour, texture, and presence of extraneous material. The samples were then cultured for bacterial growth. Observed bacterial growth was isolated and identified by a series of biochemical tests. Resistance patterns were also evaluated using the Kirby – Bauer Disk diffusion method. The chi – square test and Pearson Correlation Coefficient were used to establish statistical significance.

**Results:**

Approximately 33.1% of the total faecal samples tested were positive for enteric pathogens. *Shigella* spp. demonstrated resistance to erythromycin (91.7%), doxycyclin (83.3%), ampicillin (82.1%), cotrimoxazole (73.1%), minocycline (66.7%) and cefuroxime (54.2%). *Campylobacter jejuni* also exhibited resistance to erythromycin (87.5%), doxycyclin (75%), ampicillin (73.7%), cotrimoxazole (73.3%) and minocycline (68.8%).

**Conclusions:**

The resistance patterns of *Shigella* spp. and *Campylobacter jejuni* reported in this study necessitates the need for a comprehensive multiregional investigation to evaluate the geographical prevalence and antimicrobial resistance distributions of these microorganisms. These findings also support the need for the discovery and development of effective therapeutic alternatives.

**Trial registration:**

Retrospectively registered. Certificate No. 00762

## Background

Globally, diarrhoeal diseases is the second most common cause of death in children under five years of age. Each year, about 2.4 million children under five years of age die from diarrhoeal diseases. The highest mortality from diarrhoeal diseases occur in developing countries [1]. In Kenya, diarrhoea is the third most common cause of mortality and morbidity with a case fatality of up to 21% in children < 5 years and about 50–60% of cases are caused by bacterial pathogens [2]. Recent surveys indicate that of the 165 million cases of diarrhoea caused by *Shigella* that occur annually, 99% occur in developing countries with 69% in children under five years of age [3]. It has also been determined that *C. jejuni* is emerging to be one of the most common causes of human diarrhoeal disease and the leading cause of bacterial gastroenteritis in humans worldwide [4].

Although most diarrhoeal diseases are self-resolving and should not be treated with antimicrobial agents, it’s still significant that the control of fecal-orally transmitted pathogens is inadequate in many developing countries, in particular, in sub-Saharan Africa, leading to non-empirical selection of antibiotics. Studies reveal that acquired resistance to antimicrobial drugs is becoming more prevalent among enteric pathogens in this region. Improved antimicrobial drug stewardship is an often cited, but inadequately implemented [5]. Resistance to antimicrobials is a growing crisis in clinical medicine. In 2017, the WHO published a list of bacteria where new antibiotics to tackle them are needed urgently and grouped them according to their priority as critical, high, and medium [6]. The use of antibiotics for the successful treatment of infectious diarrhoea has been severely compromised due to the emergence of multidrug-resistant bacteria, a problem that has been acknowledged on a global scale [1]*.* Currently, there are virtually no antibiotics for the treatment of infections caused by some multidrug-resistant bacterial strains [7, 8]. Over the years, sulphonamides, tetracycline, ampicillin and trimethoprim/sulphamethoxazole have been effective drugs, only to become impotent in the face of emerging resistance [1]. One class of antibiotics whose clinical efficacy is particularly diminished due to an increase in the prevalence of resistant bacteria are the aminoglycosides such as streptomycin, gentamicin, tobramycin, kanamycin and amikacin [9]. Previously, a single dose of the quinolones: norfloxacin and ciprofloxacin were effective in treating shigellosis. Nowadays, these quinolones are less effective and newer quinolones or other classes of antibiotics such as cephalosporin derivatives and azithromycin are recommended for treatment [10]. The aminoglycosides are highly effective broad-spectrum antimicrobial agents; however, their efficacy has diminished due prevalence of aminoglycoside resistance enzymes in Gram-negative pathogens, the adenylyl transferase ANT (2″)-la, that confers resistance to gentamicin, tobramycin, and kanamycin [1].

Initial rates of resistance to new drugs are normally on the order of 1% but with the current use of antibiotics there has been an increase in the number of either inherent or acquired resistance elements that within 8–12 years after wide-spread use, strains resistant to multiple drugs become widespread [11]. Tafa et al.*,* observed that 30% or more hospitalized patients were treated with one or more courses of antibiotic therapy [12]. With such practices, the inevitable consequence of widespread and injudicious use of antibiotics has seen the emergence of antibiotic-resistant pathogens, resulting in a serious threat to global public health, as there now remains fewer, or even sometimes no effective antimicrobial agents available for the treatment of infections caused by these bacteria [13].

The aim of the current study was to determine the prevalence of antibiotic resistance as well as patterns in sensitivity profiles of *Shigella* species and *Campylobacter jejuni* isolated from faecal samples of children under five years of age presenting with diarrhoea at Kapsabet County Referral Hospital.

## Methods

### Study design and site

This hospital based cross-sectional study was conducted for 6 months at the Kapsabet County Referral Hospital in Nandi County, Kenya. The Kapsabet County Referral Hospital serves approximately 90% of the infants and children under the age of five. According to the Kapsabet County Referral Hospital records, about 11,000 children are treated annually with 5–10% of these cases presenting with acute diarrhoea.

### Study and source population

The source population were children under five years old admitted with diarrhea from whom the study population was purposively selected during the study period.

### Sample size calculation

The sample size was calculated using a single population formula with the following assumptions; 95% level of confidence (*p* = 0.05), with proportion of target population at 89.5% and design effect of 1.96. The sample size was a total of 144 pediatric patients.

### Sample collections

There were a total of 144 faecal samples were aseptically collected from all the respondent recruited using the purposive sampling technique, during the period of study. Out of these 5 cases had at least one requirement missing and were excluded from the analysis, and thus 139 samples were subjected through the investigations thus representing 97% of the total cases observed in the areas of study.

### Data collection techniques

Face to face interview with patient’s parents/guardians was conducted using a structured (closed-ended questions) questionnaire to establish baseline demographic characteristics that included gender, age and medical history.

### Bacterial isolation and identification

Faecal samples were aseptically collected from the enrolled participants, placed into the sterile plastic tube and sealed for transportation to the laboratory for microbiological analysis. Samples were analyzed within 20 min after collection as per the WHO. Each sample was examined macroscopically for colour, texture and presence of any extraneous materials, (blood, mucus or watery). A loopful of each sample was cultured in 10 ml Selenite Faeces (SF) broth for enrichment, incubated at 37 °C for 6 h and then aseptically subcultured onto MacConkey (MAC) agar, Salmonella Shigella (SS) agar for the isolation of *Shigella* spp. while Campylobacter (CAMPY) agar was used for the isolation of *C. jejuni.* The MacConkey and Salmonella Shigella agar cultures were incubated at 37 °C, aerobically for 18 h. The Campylobacter agar cultures were incubated for 72 h at 42 °C in microaerophilic conditions generated by gas pack. The identification of typical colonies from each culture was performed with the use of conventional bacteriological tests for *C. jejuni* (Hippurate hydrolysis and Commercial identification system) (Api CAMPY system) to confirm and differentiate at the species level. NLF colonies, growing from MAC and SS were selected and subjected to Selected Biochemical Tests (Triple Sugar Iron (TSI), Hi-IMViC/Hi assorted tests (KB001 and KB002 [HIMEDI-INDIA]), Identification of bacteria species was performed according to WHO recommendations [14]. For each successfully identified isolate, antibiotic susceptibility test was conducted using eleven antibiotic disks specifically ampicillin (10 μg/disc), ciprofloxacin (30 μg/disc), doxycycline (10 μg/disc), erythromycin (15 μg/disc), gentamicin (10 μg/disc), nalidixic acid (30 μg/disc), cefuroxime (30 μg/disc), cotrimoxazole (25 μg/disc), norfloxacin (30 μg/disc), ceftriaxone (30 μg/disc) and minocycline (30 μg/disc)) on Mueller-Hinton agar petri dishes by the Kirby – Bauer Disk diffusion method as per the recommendation of Clinical and Laboratory Standards Institute (CLSI) [15–18]. Zones of growth inhibition were compared to a current zone size on the interpretation table (with the standard cut off measurements for susceptibility resistance) and results were reported either as R (Resistant) or S (Sensitive) [19, 20].

### Data quality assurance

For quality assurance of data, the ward Nurse and clinical officers were instructed to collect data by checking on the provided data sheet. Prior training on proper interview techniques, foecal sampling, foecal processing, quality data collection techniques and adherence to code of ethics and professional conduct was conducted by the interviewer. Routine checks were conducted to ensure that all the components mentioned above were performed accurately.

### Data analysis

Data entry and cleaning were routinely performed using E.P.I 3.5.1 statistical software and then analyzed using SPSS Version 20.0. Frequencies and proportions were computed as descriptive analysis. The isolated pathogens were subjected to descriptive statistics using frequency tables and graphs. The chi-square test and Fisher exact tests were used to determine statistical significance (*p* ≤ 0.05).

## Results

### Baseline demographic characteristics

A total of 139 patients were investigated representing 97% of the total cases observed in the study area. The patients were categorized according to age into five groups with a sampling interval of 12 months. This grouping method was based on developmental stages, breastfeeding and the weaning ages. About 59% of the total cases were females while 42% of the total cases were males. Distribution of age by gender was generally the same in both gender but lowest in males in the 45–59 age categories (Table 1).

**Table 1:** Distribution of Age by Gender among the cases under study

Gender	Age – group (months)					Total
	0–11	12–23	24–35	36–44	45–59	
Gender of the child **(n%)**						
Male	15 (26.3)	19 (33.3)	10 (17.5)	10 (17.5)	3 (5.4)	**57**
Female	21 (25.6)	25 (30.5)	22 (26.8)	7 (8.5)	7 (8.5)	**82**
**Total**	**36 (25.8)**	**44 (31.7)**	**32 (23)**	**17 (12.2)**	**10 (7.2)**	**139**

### Clinical observation and bacterial isolates

Majority of the cases 85 (61%) presented with fulminant diarrhoea and intermittent vomiting while 54 (39%) presented with bloody diarrhoea. About 94 (68%) cases of diarrhoea samples were mucoid, 19 (14%) were bloodied while 26 (18%) cases were watery. Bacterial isolations for *Shigella* spp. were statistically associated with bloody diarrhoea and *Campylobacter jejuni* correlated significantly (*p* = 0.0001).

### Faecal isolates

*Shigella* spp. was detected in 50% of bloody diarrhoeal stool samples and 25% of the mucoid stool samples. However, *C. jejuni* was only detected in the mucoid stool samples.

*Shigella* spp. was the most common pathogen isolated with a frequency of 28 (20.1%) isolates followed by *C. jejuni* with a frequency of 18 (12.9%) (Table 2). Bacterial pathogens were not detected in 82 samples (58.9%), while 11 samples (10.9%) produced at least single colonies of non-pathogenic bacteria on MAC agar.

**Table 2:** Diarrhoeal types in relation to bacterial pathogen isolated

	Bacteria Isolated Frequency & percentage (%)		Total
	***Shigella*** spp.	***C. jejuni***	46/139
**Diarrhoea type**			
**Watery**	7 (25)	4 (22.2)	**11/26**
**Bloody**	14 (50)	0 (0)	**14/19**
**Mucus+**	7 (25)	14 (77.8)	**21/94**
**Total isolates**	**28 (20.1)**	**18 (12.9)**	**46/139**

Further cross tabulation of age-group against bacteria pathogens isolated in the study, and controlling for gender (Table 3) found *Shigella* spp. was more prevalent at both age age-groups of 24–35 months and 12–23 months, indicating a high frequency of *Shigella* infections. The peak incidence of *C. jejuni* infection (33.3%) associated with diarrhoea was observed in age categories of 12–23 months, while *Shigella*’s peak infection (39.3%) was in the age categories of 24–35 months.

**Table 3:** Proportional distribution of pathogens in relation to age and gender category

Gender of the child	*Shigella* spp.	*C.jejuni*	Total
Male			
Age			
0–11	1 (8.3)	5 (50)	6 (27.3)
12–23	3 (25)	3 (30)	6 (27.3)
24–35	6 (50)	1 (10)	7 (31.8)
36–47	1 (8.3)	1 (10)	2 (9.0)
45–59	1 (8.3)	0 (0)	1 (4.5)
Sub-Total	**12 (21.1)**	**10 (17.5)**	**22**
Female			
Age			
0–11	3 (18.8)	0 (0)	3 (12.5)
12–23	5 (31.2)	3 (37.5)	8 (33.3)
24–35	5 (31.2)	3 (37.5)	8 (33.3)
36–47	1 (6.3)	2 (40)	3 (12.5)
45–59	2 (12.5)	0 (0)	2 (8.3)
Sub-Total	**16 (19.5)**	**8 (9.7)**	**24**
Total			
Age			
0–11	4 (14.3)	5 (27.8)	9 (19.6)
12–23	8 (28.4)	6 (33.3)	14 (30.4)
24–35	11 (39.3)	4 (22.2)	15 (32.6)
36–47	2 (7.1)	3 (16.7)	5 (10.9)
45–59	3 (10.7)	0 (0)	6 (27.3)
**Total**	**28 (20.1)**	**18 (12.9)**	46 (33.1)

A strong negative significance relationship was established (r = − 0.502, *p* = 0.003) between males and bacterial isolates in these age categories. In females, there was no statistical significance established between the age age-group and bacterial isolates (r = − 0.110, *p* = 0.505), indicating no observable difference in age-group and pathogen isolated. An overall significant relationship of r = − 0.289 was observed, which was significant at 95% level with *p* = 0.014. This indicated that, as the age increases, the number of isolates decreases significantly.

On the correlation of gender with the diarrhoeal etiological agent, female dominated for all agents (r = 0.51) indicating no statistical significance between gender and the presence of bacterial pathogens. (*p* = 0.592).

### Antimicrobial susceptibility testing (AST)

Antibiotic inhibition zones were compared with current reference inhibition zones for each bacterial isolate within the limits [16, 20]. *Shigella* and *C. jejuni* were resistant to more than 50% of the test antimicrobials used (Table 4). I Isolates of *C. jejuni and Shigella spp.* were highly susceptible to ciprofloxacin. *Shigella* spp. also showed high resistance to ampicillin (82%), cotrimoxazole (73%), erythromycin (91.7%), cefuroxime (54.2%), minocycline (66.7%) and doxycycline (83.3%) (Table 4).

**Table 4:** Resistance of *Shigella* spp. and *Campylobacter jejuni* against eleven antibiotics

Bacteria	% Antimicrobial resistance										
	Antibiotic										
	AMP	CIP	COT	CTZ	CXM	DCN	ERY	GEN	MI	NA	NOR
*Shigella*	82.1	29.9	73.1	30.8	54.2	83.3	91.7	14.8	66.7	40.7	34.6
*C. jejuni*	73.7	0	73.3	40.9	6.3	75	87.5	36.8	68.8	36.8	6.2

*Legend*: *AMP* Ampicillin, *CIP* Ciprofloxacin, *COT* Cotrimoxazole, *CTZ* Ceftriaxone, *CXM* Cefuroxime, *DCN* Doxycyclin, *ERY* Erythromycin, *GEN* Gentamycin, *MI* Minocycline, *NA* Nalidixic acid, *NOR* Norfloxacin

Chi-square (χ2) value of 252.4, *p* = 0.0001 indicated that susceptibility patterns demonstrated in this study were statistically significant in relation to the bacterial isolates.

## Discussion

The appropriate treatment of diarrhoeal diseases includes fluid and electrolyte administration. Antimicrobial drug therapy is indicated for children with acute infectious gastroenteritis [17, 20]. High isolation rates of *Shigella* spp. (20.1%) were found in children < 5 years of old indicates that the bacterium is a common etiological agents implicated in diarrhoea in the area of study under focus. The categories’ frequency variations for *Shigella* spp. and *C. jejuni* may be explained by differences in age, socio-economic development level, geography, but could also be related to levels of sanitation and individual hygiene, contamination of food or water among other environmental factors. In the current study, *Shigella* species was detected almost twice as much as *C. jejuni* validating previous reports revealing that shigellosis was prevalent in rural communities [21]. Records of higher prevalence for *Shigella* species equally replicates with reports of an increasing trend in the prevalence of *Shigella* infections in sub-Saharan countries in the last ten years [22, 23]. High isolation rates of *C. jejuni* (12.9%) were in agreement with those reported from Kolkata, Algeria [7] and Burkina Faso [24], but also closely compares with studies conducted in South Africa (20.3%), and Kenya (16.4%). In other studies, Nigeria reported the highest prevalence (62.7%) followed by Malawi (21%) [25]. The differences in age groups may also be the possible explanations for the variation of the results. On the other hand, lower rates of isolation were reported from some other African countries like Uganda (9.3%), Madagascar (9.7%), and Mozambique (1.7%). Compared with the report from Vhembe district of South Africa (24.9%), the isolation rate of the current study is lower, due to culture medium used to facilitate the isolation of antibiotics sensitive *Campylobacter* strains. This level of isolation for *Campylobacter jejuni* was most probably associated with contact with domestic animals among the study subjects which probably transferred resistant strains of the organisms to children through direct contact or environmental contamination [26]. Slightly more than half (58.9%) of the patient stool samples did not yield any bacterial isolates, perhaps due to other diarrhoeal causal agents such as viruses that were not considered during the study, and/ or individual parents/guardians not including a full disclosure of antibiotic use prior to enrolment in the study.

Our results showed that there was no significant difference between infection cases in children below and above12 months of age (*p* = 0.176). This finding is similar to the 46% prevalence rate earlier reported in Ifakara, Tanzania and Mulago, Kampala both of which agree with the position of the World Health Organization that the vast majority of enteric diarrhoeal cases occur in Asia, Africa and Latin America, where waterborne diseases were highly prevalent because of inadequate supply of potable water to the public with concomitant poor environmental and personal hygiene [7, 8, 27, 28].

Results of our study showed that the number of isolations for *Shigella* increased with age and reached significant percentages in the ≥12 and ≤ 24 months age-group for both male and female children. This could be partially due immune system inability to mount an effective immune response and also the risk of placing contaminated fingers and fomites in the mouth is increased due to physiological phenomenon like crawling. The lower shigellosis rate among children under the age of 1 – year – old may be attributed to the protective immune properties of breast milk or the exclusion of *Shigella*-contaminated foods from their diet. The increased number of shigellosis cases seen over the age of 1, when most children are no longer breast feeding likely reflects the lack of natural anti- *Shigella* immunity of recently weaned children [1]. The infections observed in the 0–11 months’ group might be explained by possible unhygienic food preparation, food storage and feeding of infants as weaned foods get exposed to contamination. On the other hand, less diarrheal infection was observed with increasing age groups which is consistent with other studies, and this might reflect on possible poor immunity among children invariably due to under nutrition increases susceptibility to diarrheal diseases [2, 8, 9].

About 60% of the bacterial isolates under focus were multiple antibiotic resistant types, which is in consonance with the detection of multiple-resistant *Shigella* strains in Africa, England and Asia [29]. Cotrimoxazole is a drug often used for empirical therapy of diarrhoeal diseases, extensive use of this drug may have led to occurrence of resistant *Shigella* species. In this study Shigella spp. showed a resistant of 73% to Cotrimoxazole. Previous reports have shown a 92 to 95% resistant levels to Cotrimoxazole [30]. C*ampylobacter jejuni* was resistant to more than five antibiotics used in this study with a resistance rate of > 50%, indicating a high degree of multiple drug resistance.

The fact that *Shigella* spp. showed high resistance levels for ampicillin (82%), cotrimoxazole (73%), erythromycin (91.7%), minocycline (66.7%) and doxycycline (83.3%), strongly supports that these antibiotics should not be recommended for the treatment of shigellosis. The resistance to ampicillin in Gram-negative bacteria can be explained by the fact that it is primarily mediated by *β*-lactamases, within an intergron which hydrolyse the *β*-lactam ring and thereby inactivate the antibiotic [1, 12, 31, 32]. In East Africa and other African countries, Denmark and Greece low resistance to nalidixic acid and no resistance to ciprofloxacin has been observed in *Shigella* isolates. Cotrimoxazole is the drug of choice for shigellosis treatment, the resistance pattern in our study could be explained by the presence of a *dhfr Ia* gene previously described in *Shigella* and considered the most common dihydrofolate reductase gene in the genus [26, 33, 34]. Low resistance to ciprofloxacin, norfloxacin, ceftriaxone and cefuroxime by *Shigella* spp. indicates that these drugs may be more effective therapeutic alternatives and further supports the current use of ciprofloxacin, ceftriaxone and nalidixic acid [35, 36]. This phenomenon is critical considering the numerous factors that may contribute to resistance by pathogens causing gastroenteritis in developing countries like Kenya. These include frequent overuse, misuse and factors related to the potency and quality of antimicrobials and the distribution of resistant strains [10]. The marked increase in multidrug resistance to first line drugs especially those recommended for the treatment of bacillary dysentery according to WHO [1], and decreased resistance to the newer regimens, ciprofloxacin (7.7%) and norfloxacin (28.6%), may be as a result of a decrease in the use of non-fluoroquinolone agents such as nalidixic acid, in favour of fluoroquinolones for the treatment of both gastroenteritis and undefined febrile illnesses [10]. In addition, this study established a moderate increase in resistance to ceftriaxone, which contrasts with increasing rates of resistance and emergence of extended spectrum β-lactamase–resistant (ESBL) organisms elsewhere in Africa [37]. Approximately 6.2% of *C. jejuni* isolates were resistant to norfloxacin, though negligible, points to the emergence of resistance. Comparisons can be made with an increase in cephalosporins resistance rates from 0 to 84% recorded in Thailand and Nigeria as result of inappropriate and indiscriminate use of that class of drugs [7, 9]. C*ampylobacter jejuni* was resistant > 50% of the panel of antibiotics used indicating multiple drug resistance, an appearance which would be a worrying concern globally.

The high percentage of multiple drug resistant *Campylobacter jejuni* to most frequently used antibiotics may be due to uncontrolled use of antibiotics such as self – medicating and access to drugs without prescription [25, 38]. Most of the time, acute gastroenteritis due to *Campylobacter* species will be treated empirically with fluoroquinolones and macrolides. Although the rate of *Campylobacter* spp. resistance to these drugs is increasing in the world, the incidence is higher in developing countries. Use of these drugs for infections other than gastroenteritis and self – medicating is often the cause of resistance in developing countries. In developed countries, resistance is due to their use in food animals and travel to developing countries [26]. Ciprofloxacin resistance among *C. jejuni* in this study may be linked to possible vertical evolution. Mutations in the *gyrA* gene have also been reported to cause fluoroquinolone resistance in *C. jejuni* isolates [23, 35].

The problem of antimicrobial drug resistance is not unique to Kapsabet County Referral Hospital. It is particularly critical in many developing countries where frequent illnesses coupled with ready access to unregulated antibiotics diminishes the value of these agents for those patients who actually need them. We found a high frequency of antimicrobial resistance of *Shigella* spp. and *C. jejuni* to commonly used antibiotics such as ampicillin (82.1 and 73.7%) cotrimoxazole (73.1 and 73.3%) and erythromycin (91.7 and 87.5%). This finding is similar to what has been recently described in children from Vietnam, Central Africa, Tanzania, México, Argentina and Mozambique, where high levels of erythromycin and ampicillin resistance was observed for all the tested pathogens [14, 39]. This is also in agreement with an earlier report of a worldwide occurrence of drug resistant enteric pathogens, a development traceable to inaccurate diagnosis and inappropriate use of these drugs in the treatment of infections [21, 40].

## Conclusions

Amidst the emerging increase of *Campylobacter jejuni* infections in rural Kenya, further and more comprehensive multiregional investigations should be conducted to determine the geographic prevalence and antimicrobial resistance distributions of these microorganisms and many more as causal bacterial agents of diarrhoeal diseases. The presence of multidrug resistant *Campylobacter jejuni* and *Shigella* spp. present among < 5 year children at Kapsabet County Referral Hospital are alarming as most of the first-line and commonly affordable antimicrobial agents like cotrimoxazole, erythromycin and ampicillin were found not to be efficacious of these tested pathogens, thus limiting the therapeutic options in rural-urban areas with limited access of second-line treatment options such as ciprofloxacin and norfloxacin.

## Data Availability

The datasets used and/or analysed during the current study are available from the corresponding author on reasonable request.

## Abbreviations

WHO: World Health Organization
*C. jejuni*: *Campylobacter jejuni*
*E. coli*: *Escherichia coli*
spp.: Species
SF: Selenite Faeces
MAC: MacConkey
SS: Salmonella Shigella
CAMPY: Campylobacter
NLF: Non-lactose fermenters
TSI: Triple Sugar Iron
CLSI: Clinical and Laboratory Standards Institute
ESBL: Extended spectrum β-lactamase–resistant
AMP: Ampicillin
CIP: Ciprofloxacin
COT: Cotrimoxazole
CTZ: Ceftriaxone
CXM: Cefuroxime
DCN: Doxycyclin
ERY: Erythromycin
GEN: Gentamycin
MI: Minocycline
NA: Nalidixic acid
NOR: Norfloxacin
